# Resveratrol elicits anti-colorectal cancer effect by activating *miR-34c*-KITLG in vitro and in vivo

**DOI:** 10.1186/s12885-015-1958-6

**Published:** 2015-12-16

**Authors:** Shu Yang, Wenshuai Li, Haimei Sun, Bo Wu, Fengqing Ji, Tingyi Sun, Huanhuan Chang, Ping Shen, Yaxi Wang, Deshan Zhou

**Affiliations:** Department of Histology and Embryology, School of Basic Medical Sciences, Capital Medical University, Beijing, 100069 P. R. China; Beijing Key Laboratory of Cancer Invasion and Metastasis Research, Beijing, 100069 P. R. China; Cancer Institute of Capital Medical University, Beijing, 100069 P. R. China

**Keywords:** Colorectal cancer, KITLG, *MiR-34c*, Oxaliplatin, Resveratrol

## Abstract

**Background:**

Silence of the tumor suppressor *miR-34c* is implicated in the development of colorectal cancer (CRC). For the past few years, Resveratrol (Res) has been introduced to oncotherapies alone or with traditional chemotherapeutic drugs. However, the study of molecular mechanism involved in the anti-CRC effect of Res is still ongoing.

**Methods:**

The anti-CRC effect of Res alone or with Oxaliplatin (Oxa) was determined by cell viability assay, soft agar colony formation assay, flow cytometry and real-time cellular analyzer in HT-29 (*p53*^*+*^) and HCT-116 (*p53*^*−*^) CRC cell lines. Expressions of m*iR-34c* and its targets were detected by qPCR and/or western blot. To evaluate the role of *miR-34c* in anti-CRC effect by Res alone or with Oxa, m*iR-34c* was up or down-regulated by lentiviral mediation or specific inhibitor, respectively. To investigate how m*iR-34c* was increased by Res, the methylation status of m*iR-34c* promoter was detected by MSP. The tumor bearing mouse model was established by subcutaneous injection of HCT-116 cells to assess anti-CRC effect of Res alone or with Oxa in vivo. IL-6 and TNF-α in xenografts were detected by ELISA.

**Results:**

Res inhibited cell viability, proliferation, migration and invasion as well as promoted apoptosis both in HT-29 and HCT-116 CRC cells. The anti-CRC effect of Res was partially but specifically through up-regulating *miR-34c* which further knocked down its target KITLG; and the effect was enhanced in the presence of p53 probably through inactivating PI3K/Akt pathway. Besides, Res sensitized CRC cells to Oxa in a *miR-34c* dependent manner. The xenograft experiments showed that exposure to Res or Oxa suppressed tumor growth; and the efficacy was evidently augmented by the co-treatment of Res and Oxa. Likewise, *miR-34c* level was elevated in xenografts of Res-treated mice while the KITLG was decreased. Finally, Res clearly reduced IL-6 in xenografts.

**Conclusion:**

Res suppressed CRC by specifically activating *miR-34c*-KITLG in vitro and in vivo; and the effect was strengthened in the presence of p53. Besides, Res exerted a synergistic effect with Oxa in a *miR-34c* dependent manner. We also suggested that Res-increased *miR-34c* could interfere IL-6-triggered CRC progression.

**Electronic supplementary material:**

The online version of this article (doi:10.1186/s12885-015-1958-6) contains supplementary material, which is available to authorized users.

## Background

The incidence and mortality of colorectal cancer (CRC) rank the top 5 among all malignant neoplasms both in China and western countries. Except for exairesis, chemotherapy is one of the most common treatments for CRC patients, especially those who have distant metastasis. However, long-term use of chemotherapeutic drugs e.g. oxaliplatin (Oxa) can cause several side effects such as hepatotoxicity and neurotoxicity and induce drug resistance [1–3]; thus, it is hard to achieve the expectations. Nowadays, several natural compounds have been introduced in anti-tumor researches and clinical oncotherapies, either alone or combined with traditional chemotherapeutic drugs. Resveratrol (Res) is a natural polyphenolic compound rich in peanuts, red wine and grapes. Apart from the well-documented anti-inflammation and anti-oxidation effects [4, 5], Res has an anti-tumor potential in CRC and other cancers in vitro and in vivo [6–9], excitingly, without apparent side effects.

Efforts have been made to figure out how Res plays the anti-tumor role, which is of importance for the better application of Res in clinic. It has been reported that Res could induce cell apoptosis and cell cycle arrest via p53 pathway and/or caspase/cyclin-CDK pathway to achieve its anti-tumor activities [10, 11]. Identification of the role that microRNAs play in human cancer pathogenesis triggered researches in the regulation of Res on microRNA expressions. Depending on their targets, microRNAs could serve as either oncogenes or tumor suppressing genes. Accumulating evidence showed that Res decreased *miR-520 h* and subsequently suppressed tumor cell invasion and migration in lung cancer cells [12]. Res also inhibited cancer growth and metastasis of SW480 human CRC cells by inducing *miR-663* expression [13]. These observations clearly indicated that microRNAs were involved in the Res-mediated anti-tumor activities.

*MiR-34c* is suggested to be a candidate of tumor suppressing gene and epigenetically silenced in CRC [14, 15]. We recently found that over-expression of *miR-34c* induced apoptosis and inhibited proliferation and invasion in CRC cells by silencing its target, stem cell factor (SCF, also known as KITLG) [16], suggesting *miR-34c* as a promising target for the treatment of CRC patients. Besides, it has been recently raised that Res inhibited human CRC cell growth and induced apoptosis through up-regulating *miR-34a*, a homologue of *miR-34c*, implying a possible similar modulation of Res on *miR-34c* expression [17]. However, whether *miR-34c* is implicated in the Res-mediated anti-CRC effect has not yet been fully elucidated. Furthermore, how Res synergizes with Oxa in the treatment of CRC needs clarified besides its protection from the Oxa-induced hepatotoxicity and neurotoxicity [18].

In the present study, we provided evidence that Res itself could not only exert significant anti-CRC effect, but also showed a synergistic effect with Oxa in a *miR-34c* dependent manner.

## Methods

### Cell culture and reagents

Human CRC cell lines HT-29 (*p53*^*+*^) and HCT-116 (*p53*^*−*^) were purchased from the Cell Bank of Chinese Academy of Sciences. All cell lines were cultured in DMEM medium (Life Technologies, USA) supplemented with 10 % fetal bovine serum (Life Technologies) and 1 % penicillin/streptomycin (Life Technologies). Cells were grown at 37 °C in the presence of 5 % CO_2_ and treated with Res (Sigma, USA) and/or Oxa (Sigma). DMSO (Sigma) was used as control.

### Cell viability assay

Cell viability was detected by cell counting kit-8 (CCK-8, Dojindo Laboratories, Japan) according to the manufacturer’s protocol. Cells were seeded in 96-well plates at 3 × 10^3^ cells per well. 10 μL of the tetrazolium substrate was added to each well. The plates were incubated at 37 °C for 1 h and the absorbance was measured at 450 nm using Multiskan FC (Thermo Scientific, USA). All experiments were done in triplicate and repeated three independent times.

### Combination Index (CI) calculation

To assess the drug interactions of Res and Oxa, the CI value defined by median-effect analysis was calculated as follows:

$$ \mathrm{C}\mathrm{I} = {\left({\mathrm{D}}_{\mathrm{X}}\right)}_{\mathrm{Res}}/{\left(\mathrm{D}\right)}_{\mathrm{Res}} + {\left({\mathrm{D}}_{\mathrm{X}}\right)}_{\mathrm{Oxa}}/{\left(\mathrm{D}\right)}_{\mathrm{Oxa}} $$

where (D)_Res_ and (D)_Oxa_ are the doses for each drug alone that inhibits 50 % cell viability, and (D_X_)_Res_ and (D_X_)_Oxa_ are the doses for Res and Oxa in a combination that inhibits 50 % cell viability. CI < 1 indicates a synergistic effect; CI = 1, additive effect; and CI > 1, antagonistic effect [19].

### Soft agar colony formation assay

The bottom of 6-well plate was coated with 0.6 % low melting agarose (Promega, USA) and covered with 0.35 % agarose containing 1000 cells. The plates were incubated at standard incubator condition for 2 weeks. Colonies were counted under inverted phase contrast microscope (Leica DMI3000 B, Germany) in 9 randomly sampled visual fields each well by stereological technique. All experiments were done in triplicate.

### Western blot

The harvested cells were suspended in RIPA (Radio-Immunoprecipitation Assay) lysis buffer (Applygen, Beijing, China). After 10 % SDS-PAGE, the proteins were transferred onto PVDF membrane (Merk-Millipore, USA) and blocked with 5 % non-fat dry milk or 5 % bovine serum albumin (Sigma) for 1 h. The membrane was incubated with rabbit anti-KITLG (1:500, Abcam, UK), mouse anti-p53 (1:1000, Cell Signaling Technology, USA), rabbit anti-Akt (1:1000, Cell Signaling Technology) or rabbit anti-p-Akt (1:2000, Cell Signaling Technology) primary antibody at 4 °C overnight. Then, the membrane was incubated with HRP-conjugated secondary goat anti-mouse IgG (1:2000, Santa Cruz, USA) or goat anti-rabbit IgG (1:2000, Santa Cruz) for 1 h at 25 °C. The proteins were detected using ECL chemiluminescence (Thermo Scientific) and viewed in Fusion FX Vilber Lourmat (France). A mouse anti-actin (1:4000, Santa Cruz) antibody was used as an internal control.

### Real-time monitoring of cellular proliferation, migration and invasion

We employed real-time cellular analyzer (RTCA, ACEA Biosciences, USA) to monitor cellular proliferation, migration and invasion as we previously described [16]. Briefly, for the proliferation assay, the cells were seeded in E-plates at a density of 8000 cells/well and the cell index (CI) was automatically recorded every 15 min. For the migration assay, the cells were seeded in the upper chambers of CIM-plates in serum-free medium at a density of 20,000 cells/well. The bottom chambers of CIM-plates were filled with serum-containing medium to promote migration across the membranes towards the serum gradient. The CI was registered only from the cells that were capable of migrating through the membranes. The protocol for the invasion assay was identical to that for the migration assay, except that the upper chambers were loaded with 30 μl of 5 % Matrigel (BD Biosciences, USA) solution to create a 3D biomatrix film in each well prior to cell seeding. Four independent experiments were performed respectively. The slope of CI curve was analyzed.

### RNA extraction and qPCR

The total microRNA was extracted using miRNApure Mini Kit (CWBiotech, Beijing), according to the manufacturer’s instruction. Reverse transcription was performed using Taqman microRNA RT Kit (Life Technologies) and Taqman microRNA Assay with specific stem-loop primers. Real-time PCR was performed using Taqman Universal Master Mix II (Life Technologies). The reactions were incubated at 95 °C for 10 min, followed by 40 cycles of 95 °C for 15 s and 60 °C for 1 min in ABI 7500 real-time PCR system. Results were normalized to the internal control, *RNU6B*.

The total RNA was extracted using TRIzol reagent (Life Technologies) according to the manufacturer’s instruction. Reverse transcription reactions were performed using High Capacity RNA-to-cDNA Kit (Life Technologies). Real-time PCR was performed in ABI 7500 real-time PCR system using SYBR Green PCR Master Mix (Life Technologies). The primers are listed in Table 1. The reactions were incubated at 95 °C for 10 min, followed by 40 cycles of 95 °C for 15 s and 60 °C for 1 min.

**Table 1:** Primers

*KITLG*	Forward	CAGAGTCAGTGTCACAAAACCATT
	Reverse	TTGGCCTTCCTATTACTGCTACTG
*E-cadherin*	Forward	TGCCCAGAAAATGAAAAAGG
	Reverse	GTGTATGTGGCAATGCGTTC
*PTEN*	Forward	GCTGTGGTTGCCACAAAGTGCC
	Reverse	GCAGGTAGAAGGCAACTCTGCCA
*HOXB3*	Forward	AAAGGCACAAAACACGTTCC
	Reverse	GGATCTCTCACCATCCCTGA
*FGFR1*	Forward	CCCGTAGCTCCATATTGGACA
	Reverse	TTTGCCATTTTTCAACCAGCG
*Pim*	Forward	GAGAAGGACCGGATTTCCGAC
	Reverse	CAGTCCAGGAGCCTAATGACG
*GAPDH*	Forward	AGAAGGCTGGGGCTCATTTG
	Reverse	AGGGGCCATCCACAGTCTTC
Unmethylation	Forward	TTTTTATTTGTTTTGTTTTGTGTTTGTTTTG
	Reverse	CCTAAAACTAACTCTCTCAACCCCA
Methylation	Forward	ATTCGTTTCGTTTCGCGTTCGTTTC
	Reverse	CTAAAACTAACTCTCTCGACCCCG

All reverse transcription reactions included no-template controls, and all PCR reactions were run in triplicates. Relative microRNA or mRNA expression was determined using the comparative C_T_ (2^-ΔΔCt^) method.

### Flow cytometry

For cell apoptosis analysis, AlexaFluor^®^ 488 Annexin V/Dead Cell Apoptosis Kit (Life Technologies) was used according to the manufacturer’s instruction. In Brief, cells were seeded in 6-well plates and 1 × 10^5^ cells were resuspended in annexin binding buffer, after which annexin V and propidium iodide (PI, 100 μg/mL) were added and incubated at 25 °C for 15 min in the dark. Cell apoptosis was analyzed in Coulter EPLCS XL (Beckman Coulter, USA). All experiments were done in triplicate.

For cell cycle analysis, 1 × 10^6^ cells were fixed with 70 % ethanol for 2 h at 4 °C, followed by the treatment with phosphate buffered saline containing RNase (50 μg/mL) for 30 min at 37 °C. Cells were stained with PI (500 μg/mL, Sigma) for 30 s in the dark. Cell cycle kinetics were analyzed by measuring the DNA contents in Coulter EPLCS XL. All experiments were done in triplicate.

### Over-expression of *miR-34c* by lentiviral mediation

The full length of *pre-miR-34c* was chemically synthesized and introduced into GV217 lentiviral vector (GeneChem, Shanghai, China) in the unique EcoRI site to construct a lentivirus encoding *miR-34c* (Lv-*miR-34c*). The Lv-*miR-34c* or its control, Lv-NC, was transfected into CRC cells seeded in 6-well plates when reaching 30 % confluence. After 3 days, the infectious efficiency was evaluated by observing the EGFP-expression with an inverted phase contrast microscope (Leica DMI3000 B, Germany).

### *MiR-34c* knockdown

For knockdown of *miR-34c*, the specific *miR-34c* inhibitor was purchased from Ribobio (Guangzhou, China). The inhibitor or its control, inhibitor-NC, was transfected into CRC cells using riboFECT™ CP Transfection Kit (Ribobio) according to the manufacturer’s instruction.

### Methylation Specific PCR (MSP)

The genomic DNA of CRC cells was extracted using QIAamp® DNA Mini Kit (Qiagen, USA). 200 ~ 500 ng DNA was subject to bisulfite conversion using EZ DNA Methylation-Gold™ Kit (Zymo Research, USA). The methylation-sensitive PCR was performed using Platinum Taq DNA Polymerase (Life Technologies). The PCR reaction conditions consisted of an initial incubation at 94 °C for 2 min, followed by 35 cycles of 94 °C for 30 s, 55 °C for 30 s and 68 °C for 1 min using verity 96-well thermo cycler (Applied Biosystems). The primers are listed in Table 1. The PCR products were electrophoresed in 0.75 % agarose gel, and visualized by uitraviolet illumination.

### Xenograft in BALB/c nude mouse

In order to determine the in vivo anti-CRC effect of Res, the CRC cell xenograft in BALB/c athymic nude mice (3–4 weeks old) were performed. Twenty-eight nude mice were purchased from the Experimental Animal Center in the Capital Medical University and housed under Specific Pathogen Free condition. 5 × 10^6^ HCT-116 cells suspended in 50 μL phosphate buffered saline were subcutaneously injected into the right armpit of the nude mice. Ten days after cell xenograft, the nude mice were randomly grouped (7 mice/group) and received Res (100 mg/kg), Oxa (10 mg/kg), Res (100 mg/kg) + Oxa (10 mg/kg) or DMSO via tail-vein injection every day for 2 weeks based on modification of previous report [18]. The body weight and tumor size were measured every other day. The tumor volume was calculated as: W^2^ × (L/2), where W represents the tumor width and L the tumor length. The experimental procedures were approved by the Institutional Review Board of the Capital Medical University.

### ELISA

The xenografts were lyzed; and the supernatants from xenografts as well as the serum of mice were collected. The concentrations of IL-6 and TNF-α was determined by ELISA according to the manufacturer’s instructions (R&D Systems, USA). The absorbance of each well at 490 nm was read using the microplate reader (Multiskan FC, Thermo Scientific). All experiments were done in triplicates.

### Statistics analysis

All statistical analysis were performed using SPSS 13.0 (Chicago, USA). Values are expressed as means ± SEM. Results were analyzed by Student’s *t* test or one-way ANOVA. The *P* value < 0.05 was considered to be statistically significant.

## Results

### In vitro anti-CRC effect of Res

**Fig. 1:**
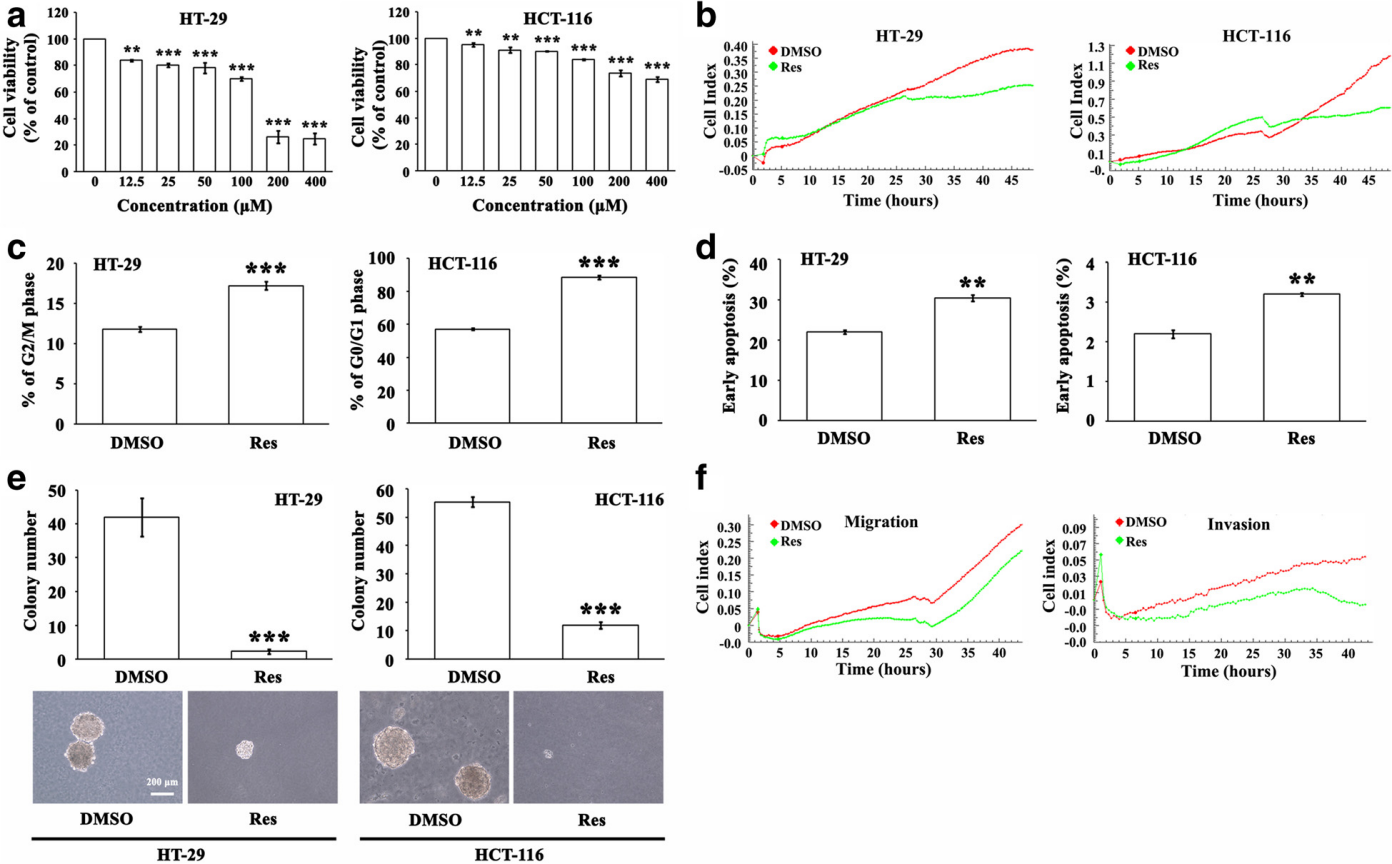
**a** Twenty-four hr-Res treatment significantly reduced CRC cell viability detected with CCK8 in a dose-dependent manner (12.5 ~ 400 μМ). ** *P* < 0.01, *** *P* < 0.001 (**b**) CRC cell growth was monitored by RTCA for 48 h in the presence of Res or DMSO. Cells treated with Res displayed lower proliferation. **c** Res induced cell cycle arrest in HT-29 cells (G2/M phase) and HCT-116 cells (G0/G1 phase) analyzed by flow cytometric analysis. *** *P* < 0.001 (**d**) Res increased cell apoptosis in CRC cells analyzed by flow cytometric analysis. ** *P* < 0.01 (**e**) Soft agar colony formation assay showed that the size and number of colonies in Res-treated CRC cells were reduced. *** *P* < 0.001 (**f**) Res inhibited the migratory and invasive capacities of HCT-116 cells monitored by RTCA

Cell viability was significantly reduced after 24 h-treatment with Res in a dose dependent manner (*P* < 0.01 ~ 0.001, Fig. 1a). Based on the results, we used 100 μM Res for HT-29 cells and 50 μM Res for HCT-116 cells in the following experiments. Importantly, it has been delineated that Res derivatives had no effect on non-tumor cells (IEC18 intestinal epithelium cells) [20]. By real-time monitoring in RTCA we found that Res decreased cell proliferation by 45.9 % in HT-29 cells (*P* < 0.05) and 57.4 % in HCT-116 cells (*P* < 0.05) (Fig. 1b). To find out the mechanism of the Res-induced proliferative suppression, we detected the cell cycle by flow cytometry. HCT-116 cells arrested in G0/G1 phase were elevated by 54.4 % (*P* < 0.001) after treated with Res, and the percentage of G2/M phase HT-29 cells were elevated by 37.1 % (*P* < 0.001) (Fig. 1c, Additional file 1: Figure S1), indicating that Res inhibited the proliferation of CRC cells by arresting cell cycle. Res as well increased apoptosis in HT-29 cells by 38.6 % (*P* < 0.01) and in HCT-116 cells by 45.5 % (*P* < 0.01) (Fig. 1d, Additional file 2: Figure S2). Effect of Res on the malignant transformation of CRC cells was examined by soft agar colony formation assay. The number and size of colonies in the Res-treated HCT-116 and HT-29 cells were obviously reduced compared with those in controls (*P* < 0.001, Fig. 1e). Besides, Res significantly suppressed cell migration by 42.2 %, (*P* < 0.01) and invasion by 51.1 % (*P* < 0.01) of HCT-116 cells observed by RTCA (Fig. 1f).

### Anti-CRC effect of Res is partially through activating *miR-34c*-KITLG axis

**Fig. 2:**
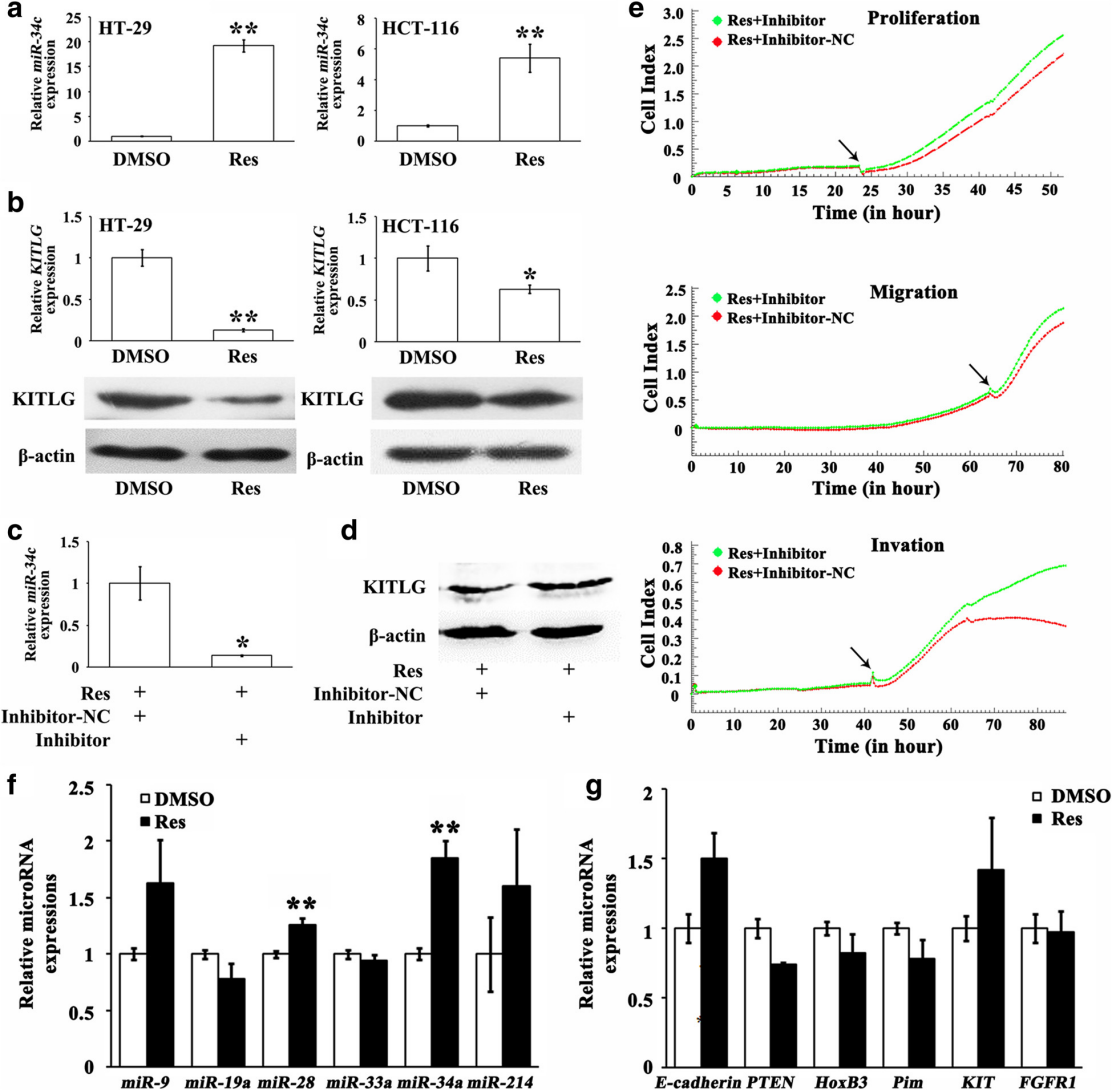
**a** Res increased *miR-34c* in CRC cells, which was more prominent in *p53* ^*+*^ HT-29 cells than in *p53* ^*−*^ HCT-116 cells. ** *P* < 0.01 (**b**) KITLG, the target of *miR-34c*, was significantly decreased at mRNA and protein levels upon the treatment of Res. * *P* < 0.05, ** *P* < 0.01 (**c**) *MiR-34c* inhibitor efficiently knocked down *miR-34c* in HCT-116 cells following 24 h-exposure to Res compared with inhibitor-NC. * *P* < 0.05 (**d**) Res-decreased KITLG protein was recovered in the presence of *miR-34c* inhibitor in HCT-116 cells; while inhibitor-NC had no effect. **e** Res-refrained proliferation, migration and invasion were reversed in HCT-116 cells in the presence of *miR-34c* inhibitor. Arrows denotes the time when *miR-34c* inhibitor or inhibitor-NC was added into the medium. **f** Res elevated *miR-28* and *miR-34a* levels in HCT-116 cells; while other microRNAs remained stable. ** *P* < 0.01 (**g**) Res did not alter the target genes of the selected microRNAs in HCT-116 cells

MicroRNAs emerge as potent regulators of numerous oncogenes and anti-oncogenes. We then asked whether the anti-CRC role of Res could be through microRNAs. We selected tumor suppressing microRNA, *miR-34c* which we have previously reported [16], to investigate the potential anti-CRC mechanism of Res. After exposure to Res for 24 h, *miR-34c* was strikingly increased 5.4 folds (*P* < 0.01) in HCT-116 cells and 19.2 folds (*P* < 0.01) in HT-29 cells (Fig. 2a), while KITLG, a target of *miR-34c* [16], was evidently decreased in these CRC cells (*P* < 0.05, Fig. 2b). To consolidate the role of *miR-34c* in the Res-mediated anti-CRC activity, we knocked down *miR-34c* in HCT-116 cells by its specific inhibitor (Fig. 2c). The inhibitory effect of Res on KITLG expression was abolished when silencing the endogenous *miR-34c* (*P* < 0.05, Fig. 2d). Interestingly, accompanied with the reduced *miR-34c* the anti-CRC effect of Res were attenuated. The Res-suppressed cell proliferation, migration and invasion were recovered by 14.6% (*P* < 0.05), 11.9% (*P* < 0.05) and 49.3% (*P* < 0.01) respectively in HCT-116 cells in the presence of *miR-34c* inhibitor (Fig. 2e). Moreover, we detected some other documented oncogenic microRNAs including *miR-9* and *miR-19a*, and tumor suppressing microRNAs including *miR-28*, *miR-33a*, *miR-34a* and *miR-214*, as well as their targets *E-cadherin*, *PTEN*, *HoxB3*, *Pim*, *KIT* and *FGFR1*, respectively [21–26], to figure out the Res-induced microRNA expression profile. Results showed that despite Res induced *miR-28* and *miR-34a* (*P* < 0.01, Fig. 2g), their respective targets *HoxB3* and *KIT* were not decreased during Res treatment (Fig. 2g), suggesting that Res probably had a specific effect on *miR-34c*-KITLG axis in CRC cells. Collectively, these results demonstrated that the anti-CRC effect of Res could be partially through activating the *miR-34c*-KITLG axis.

### Upregulation of *miR-34c* by Res is not through demethylation but p53 related

**Fig. 3:**
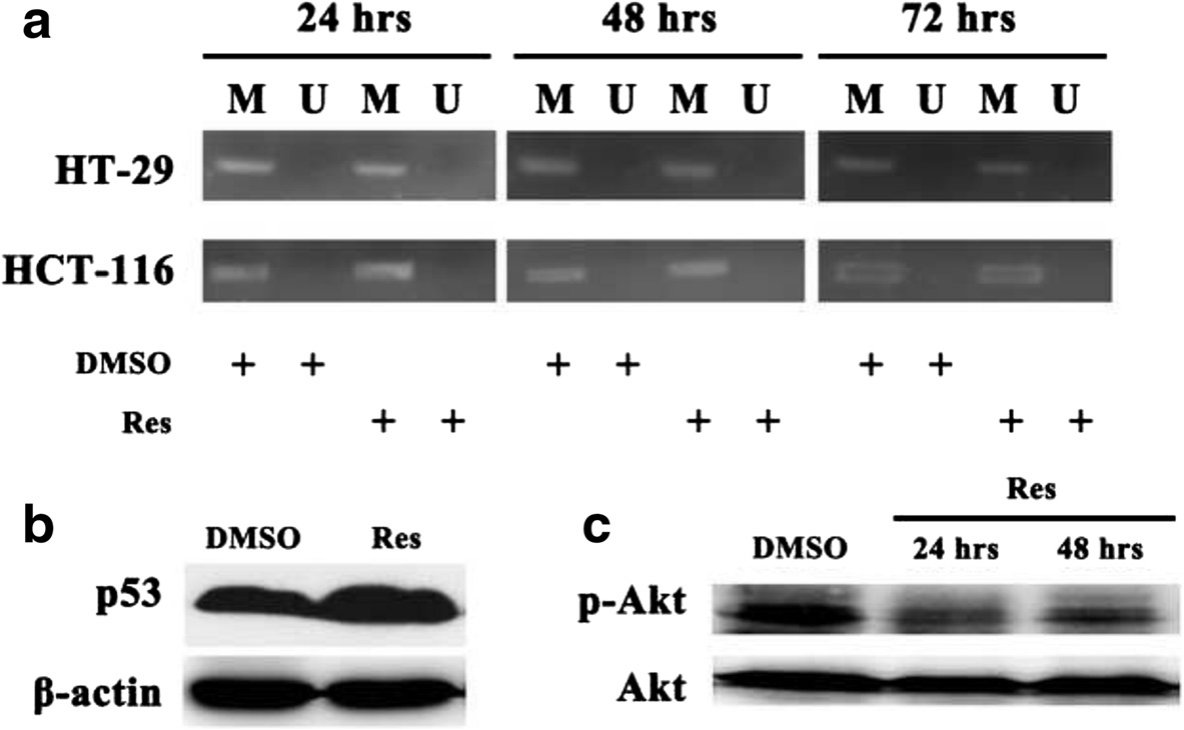
**a** The methylation state in the *miR-34c* promoter of CRC cell was not altered in the presence or absence of Res. U: unmethylation, M: methylation (**b**) Res promoted p53 expression. **c** Res inhibited phosphorylation of Akt in *p53* ^*+*^ HT-29 cells

It is well accepted that the promoter of *miR-34c* is hypermethylated in CRC tissues and cell lines, which lead to silencing of *miR-34c* [27]. So we carried out MSP to investigate whether the Res-induced *miR-34c* in CRC cells could be resulted from demethylation of the *miR-34c* promoter. Unfortunately, we did not find apparent demethylation upon the treatment of Res for 24 h, 48 h or 72 h, indicating the upregulation of *miR-34c* by Res was not through inducing demethylation of the *miR-34c* promoter (Fig. 3a).

*MiR-34c* transcription is under the control of p53 which is hypoexpressed in CRC tissues and cell lines [27]. Could p53 be involved in the Res-induced *miR-34c* in CRC cells? We used *p53*^*+*^ and *p53*^*−*^ CRC cells and found elevated *miR-34c* in both CRC cell lines, indicating that Res regulated *miR-34c* in a p53 independent way. However, Res increased p53 protein in *p53*^*+*^ HT-29 cells (Fig. 3b), and the inducement of *miR-34c* by Res was more prominent in HT-29 cells than that in *p53*^*−*^ HCT-116 cells (Fig. 2a), suggesting an involvement of p53 in the Res-induced *miR-34c* in CRC cells. Liu et al. [28] recently stated that Res inhibited CRC cell proliferation through restraining PI3K/Akt signaling that is involved in p53 degradation. In our study, the phosphorylation of Akt was also attenuated by 24 or 48 h-Res treatment, which was, however, only observed in *p53*^*+*^ HT-29 cells (Fig. 3c).

### Res sensitizes CRC cells to Oxa by up-regulating *miR-34c*

**Fig. 4:**
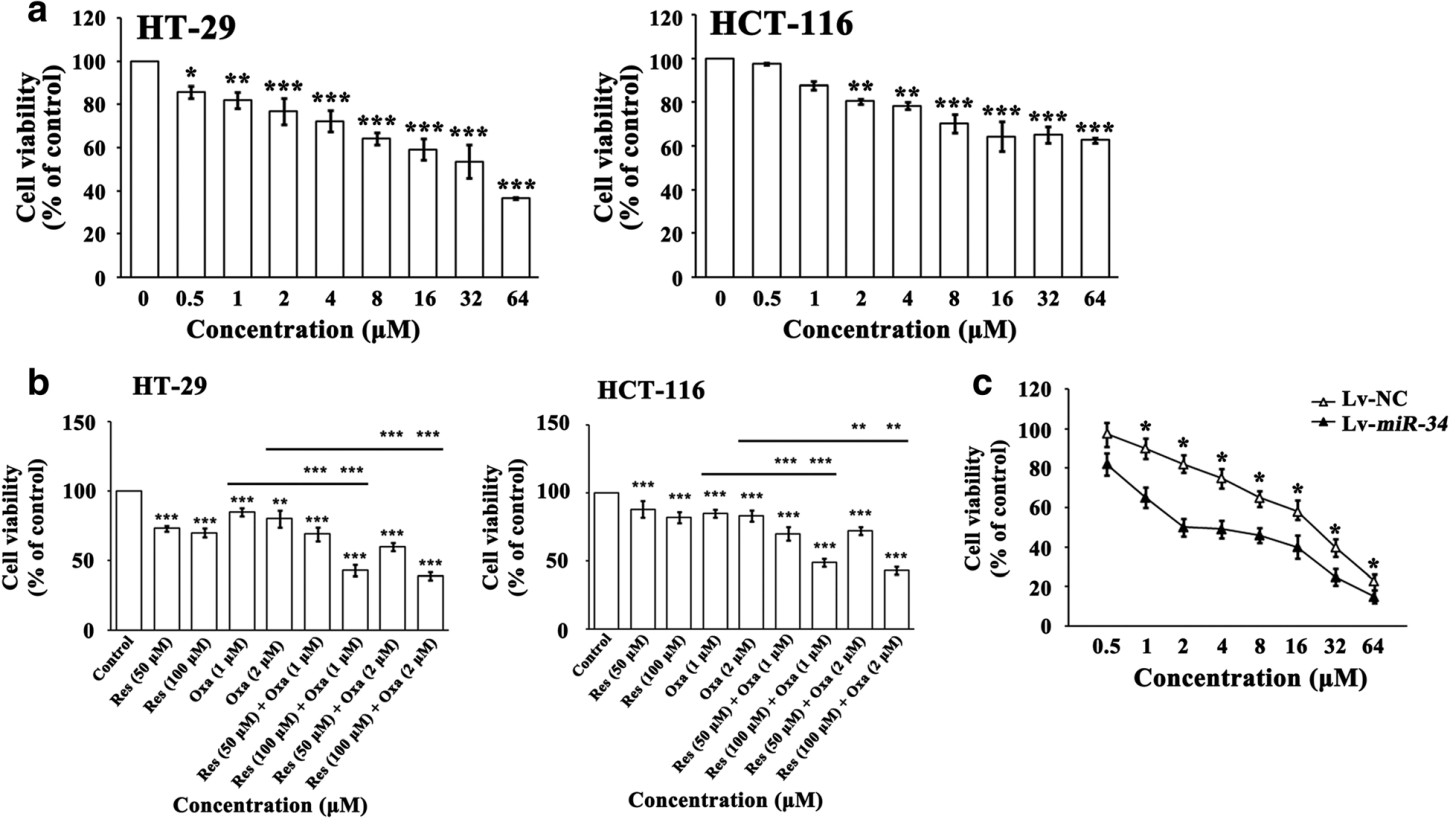
**a** Twenty-four hr-Oxa treatment evidently inhibited CRC cell viability detected with CCK8 in a dose-dependent manner (0.5 ~ 64 μМ). * *P* < 0.05, ** *P* < 0.01, *** *P* < 0.001 (**b**) Co-treatment with Res and Oxa elicited significant synergic effects on CRC viability inhibition, according to the CI values which was < 1. ** *P* < 0.01, *** *P* < 0.001 (**c**) Cell viability was deeply weaker in HCT-116 cells overexpressing *miR-34c* compared with controls in the presence of 2 μM Oxa. * P < 0.05

Oxa has been widely used in treating CRC patients. Consistent with previous studies, our result showed that Oxa significantly inhibited CRC cell viability in a dose dependent manner (*P* < 0.05 ~ 0.001, Fig. 4a). To explore whether there was a synergism of Oxa and Res, the CRC cells were treated with Res and Oxa concurrently for 24 h. Res induced potentiation of cell viability inhibition mediated by Oxa as indicated by CI analysis (CI = 0.66 in HT-29 cells and 0.27 in HCT-116 cells) (*P* < 0.01 or 0.001, Fig. 4b), suggesting that Res have synergistic anti-CRC effect with Oxa.

Next, we wished to find out the underlying mechanism of the synergistic anti-CRC effect of Res and Oxa. Since Res markedly increased *miR-34c* in CRC cells, we presumed that the up-regulated *miR-34c* could be a potential contributor. Therefore, we over-expressed *miR-34c* in HCT-116 cells by lentivirus. Upon the treatment with Oxa for 48 h, the viability of HCT-116 cells overexpressing *miR-34c* was significantly decreased compared with controls (*P* < 0.05, Fig. 4c). The result provided evidence that Res sensitized the CRC cells to Oxa chemotherapy probably through up-regulating *miR-34c*.

### In vivo anti-CRC effect of Res

**Fig. 5:**
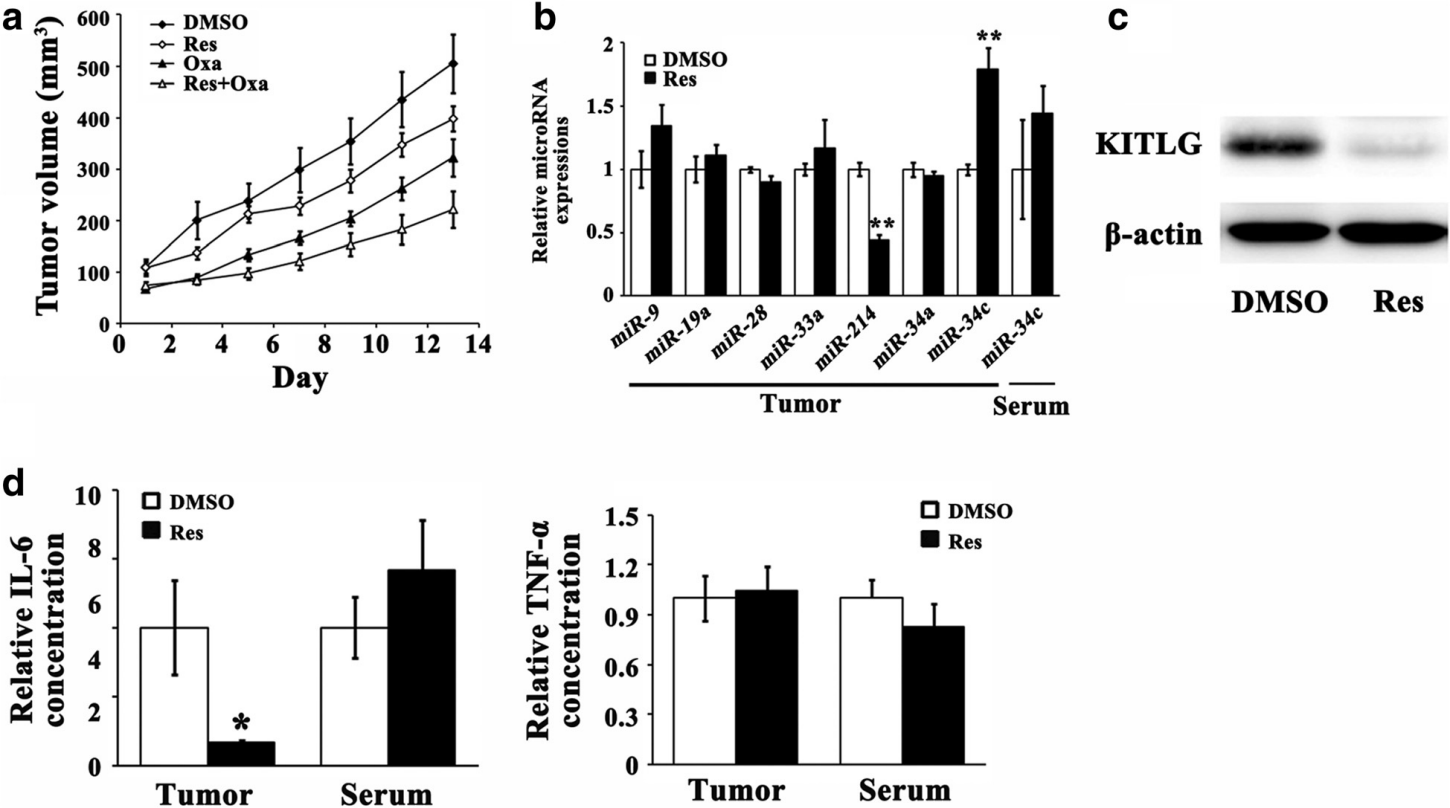
**a** Res or Oxa could significantly restrict the xenograft growth and the effect was overtly enhanced when co-treated with Res and Oxa. **b** Res increased *miR-34c* in tumors but not in serum. *MiR-214* was also increased in tumors after Res treatment. ** *P* < 0.01 (**c**) KITLG was apparently reduced in tumors of Res-treated mice. **d** IL-6 in tumors was diminished while TNF-α stayed unvarying

To qualify the anti-CRC efficacy of Res in vivo, we performed HCT-116 xenograft experiment in BALB/c nude mice. As shown in Fig. 5a and Additional file 3: Figure S3, Res or Oxa alone significantly inhibited tumor growth (*P* < 0.05); and the co-treatment of Res and Oxa elicited a clearly additive effect, indicated by the much slower tumor growth compared with Res or Oxa alone, respectively (*P* < 0.05). It was noted that 3 out of 7 xenografts vanished during the treatment of Res and Oxa. Since DMSO has chronic toxicity for animal, we measured the body weight during the animal experiment. The body weight of mice in the 4 groups did not significantly changed during experiment (Additional file 4: Figure S4). We further detected microRNA profile in xenograft tumors and/or serum; and the results showed only *miR-34c* was clearly elevated in tumors but not in serum after exposure to Res for 2 weeks, indicating that Res had a relatively specific effect on *miR-34c* expression in vivo (*P* < 0.01, Fig. 5b). Simultaneously, KITLG was down-regulated in xenografts of Res-treated mice (Fig. 5c). Interleukin-6 (IL-6) and tumor necrosis factor-α (TNF-α) are important proinflammatory cytokines produced by inflammatory cells as well as tumor cells. Clinical data revealed that IL-6 and TNF-α acted as growth factors and were associated with increased risk and advanced stages of CRC [29–32]. Our in vivo study displayed that Res decreased IL-6 secretion in tumors (*P* < 0.05) while TNF-α remained unchanged (Fig. 5d).

## Discussion

Res has been of interest in oncotherapies because of its potent anti-tumor activities and minimal side effects. To better understand the underlying mechanism of Res in treating CRC, we conducted in vitro and in vivo experiments. Consistent with previous reports, Res repressed CRC cell viability, proliferation, capacity of forming colonies, migration and invasion, as well as induced cell cycle arrest and apoptosis. Note that, Res arrested HCT-116 cells in G0/G1 phase while HT-29 cells in G2/M phase, indicating that the effect of Res on the cell cycle arrest was cell type dependent. The most interesting result was that the anti-CRC effect of Res was partially through up-regulating the tumor suppressing microRNA, *miR-34c*, which silenced its target KITLG. Besides *miR-34c*, previous studies showed that Res could increase some tumor suppressing microRNAs in CRC. Namely, Res increased *miR-663* that targeted TGF-β and potentiated the anti-CRC effect of Res [13]. *MiR-129* was suggested to participate in the anti-CRC action of piceatannol, a naturally occurring analog of Res, by targeting Bcl-2 [33]. Nevertheless, though we did detect significant increases of tumor suppressing microRNAs, *miR-28* and *miR-34a*, their respective targets, *HoxB3* and *KIT*, were not decreased, implying that *miR-28*-HoxB3 and *miR-34a*-KIT axes may not involve in the anti-CRC activities of Res. But we could not exclude the involvement of *miR-28* and *miR-34a* since they may potentiate the anti-CRC effect of Res by silencing other undetermined targets which we did not detect in this study. Fortunately, miRNA microarrays showed that Res treatment in SW480 CRC cells significantly decreased *miR-17*, *miR-21*, *miR-25*, *miR-92a-2*, *miR-103-1* and *miR-103-2* which have been shown to behave as onco-miRNAs [13]. Here, we also showed that Res did not up-regulate oncogenic *miR-9* and *miR-19a* in HCT-116 cells. It was thus inferred that the modulation of Res on the microRNA expressions was distinct: positive on tumor suppressing microRNAs and negative on oncogenic microRNAs. However, the reasons for the distinct effects of Res have been veiled yet. Taken together, we proposed that Res had a robust positive effect on *miR-34c*-KITLG axis in CRC cells and the effect was, to some extent, specific.

It’s acknowledged that the promoter of *miR-34c* is hypermethylated in CRC tissues and cell lines which lead to silencing of *miR-34c* [27]. Therefore, we considered Res-induced demethylation might be responsible for the increased *miR-34c*. However, we did not observe any demethylated bands by MSP method upon the treatment of Res, indicating demethylation did not account for the Res-induced *miR-34c*. P53 is a tumor suppressor and drives *miR-34c* transcript [27]. Could p53 conduced to the Res-stimulated *miR-34c* expression in CRC cells? Res increased *miR-34c* expression both in *p53*^*+*^ and *p53*^*−*^ CRC cells, suggesting the effect of Res on *miR-34c* expression was p53 independent. However, it was worth noting that p53 was elevated after treated with Res in HT-29 cells and, simultaneously, the inducement of *miR-34c* by Res was more prominent in HT-29 cells, indicating p53 facilitated the effect of Res on *miR-34c* expression. We also found inactivation of PI3K/Akt signaling upon the treatment of Res only in HT-29 cells, which possibly account for the up-regulation of p53 by inhibiting MDM2, a ubiquitin protein ligase controlling the degradation of p53. A recent work provided a new evidence that Res can bind directly and distinctively to *miR-33a* and *miR-122* and divergently modulate their levels in hepatic cells [34]. Whether the increase of *miR-34c* in CRC was due to the stabilization of Res deserves deep investigation. If so, we hope to find out the underlying mechanism of the distinct effects of Res on the oncogenetic microRNAs and tumor suppressing microRNAs.

Oxa is one of the first choices treating CRC patients currently; but the side effects and drug resistance caused by the long-term use interfere with the therapeutic efficiency. In this study, Res was introduced to facilitate the chemosensitivity to Oxa in CRC cells, which was probably relied on the elevated *miR-34c*. Moreover, no publications showed that Res was harmful to patients, suggesting Res could be a safe medication in preventing CRC. Lines of evidence suggested Res reversed multidrug resistance by down-regulating *MDR-1* gene in breast cancer cells [35, 36]. Wen et al. [37] addressed that PI3K/Akt signaling positively regulated *MDR-1* in CRC. According to the previous and present results, we presumed that Res-induced *miR-34c* in CRC cells might be associated with the reduced *MDR-1* or other multidrug resistance-related genes via silencing PI3K/Akt pathway, which, however, needs further investigation.

The in vivo xenograft experiment further consolidated the in vitro results. Res raised the *miR-34c* expression in tumors but not in serum, giving a clue that the *miR-34c*-inducing effect of Res was tissue specific instead of systemic. Moreover, the proinflammatory cytokine IL-6 was reduced exposure to the 2-week Res treatment. IL-6 could act as growth factor in multiple tumors by activating the oncogenic STAT3 transcription factor. Rokavec et al. [38] identified that *miR-34a/c* could target IL-6R by binding to its 3′ untranslated region (3′UTR); thereby disrupted the IL-6/IL-6R-STAT3-*miR-34a* loop. Here, we hypothesized that the IL-6-triggered CRC progression could be interfered by the Res-increased *miR-34c*.

## Conclusion

In summary, the present study revealed that Res inhibited CRC by activating *miR-34c*-KITLG in vitro and in vivo; and the effect was strengthened in the presence of p53. In addition, the up-regulated *miR-34c* by Res sensitized chemosensitivity to Oxa treatment in CRC cells.

## Additional files

Additional file 1: Figure S1. Representative histograms of the cell cycle detected by flow cytometry. (TIF 5617 kb) Additional file 2: Figure S2. Representative diagrams of the apoptosis detected by flow cytometry. (TIF 2449 kb) Additional file 3: Figure S3. HCT-116 xenografts in nude mice were collected after 2-week treatment of Res, Oxa or Res + Oxa. (TIF 3.44 mb) Additional file 4: Figure S4. There was no apparent body weight lost in any group during the animal experiment, suggesting no toxicity for mice. (TIF 2934 kb)
