# Supplementary figures and images for: Resveratrol elicits anti-colorectal cancer effect by activating miR-34c-KITLG in vitro and in vivo
Source: BMC Cancer. 2015 Dec 16;15:969. doi: 10.1186/s12885-015-1958-6 (PMC4682213; doi:10.1186/s12885-015-1958-6)

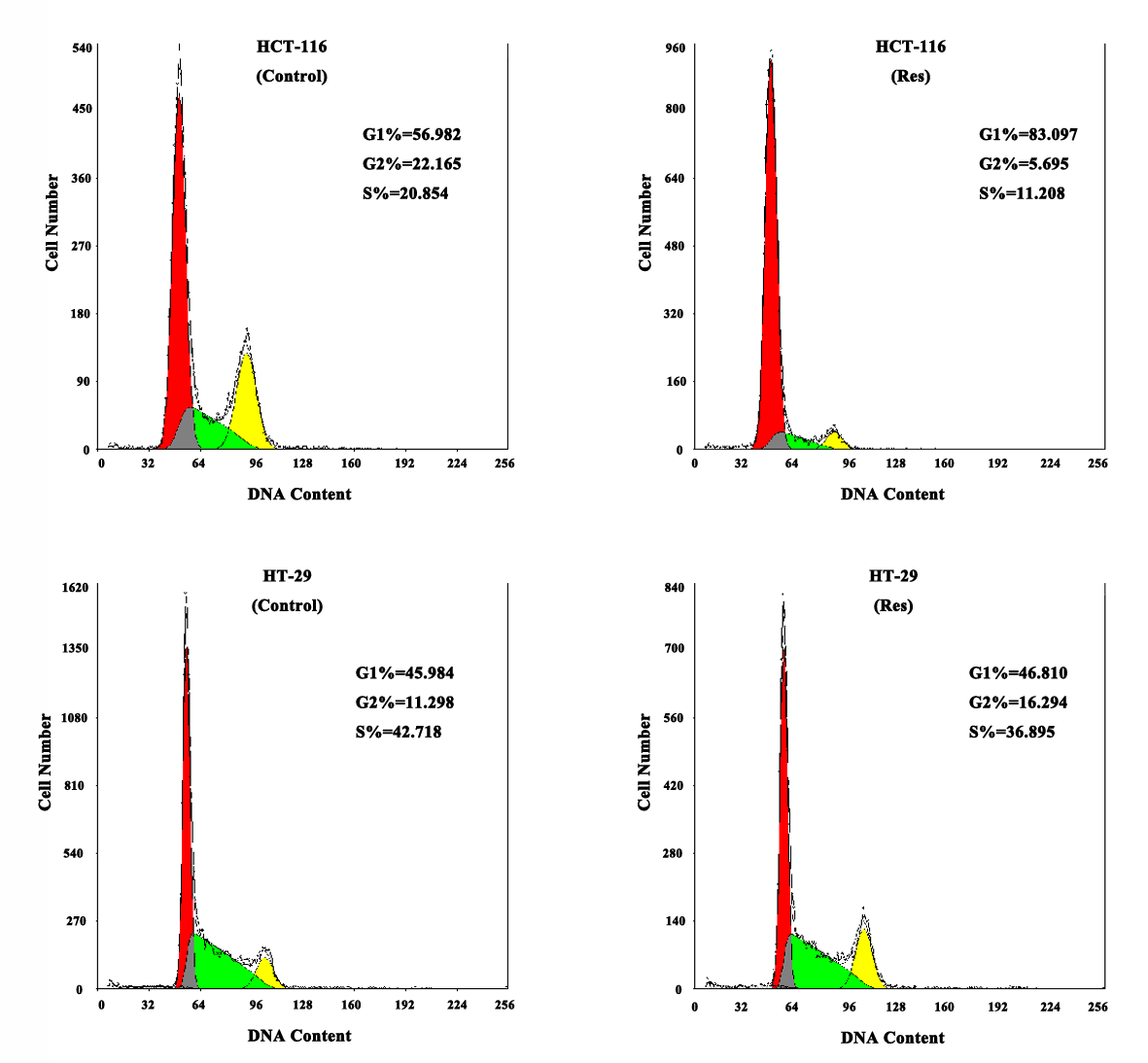

Supplement: Additional file 1: Figure S1. — Representative histograms of the cell cycle detected by flow cytometry. (TIF 5617 kb) [file 12885_2015_1958_MOESM1_ESM.tif]

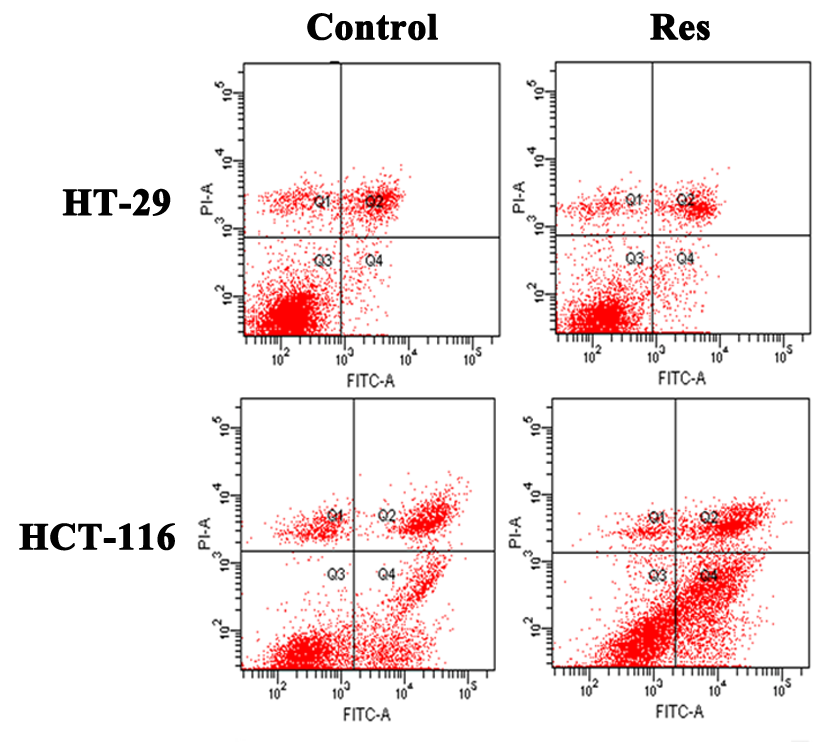

Supplement: Additional file 2: Figure S2. — Representative diagrams of the apoptosis detected by flow cytometry. (TIF 2449 kb) [file 12885_2015_1958_MOESM2_ESM.tif]

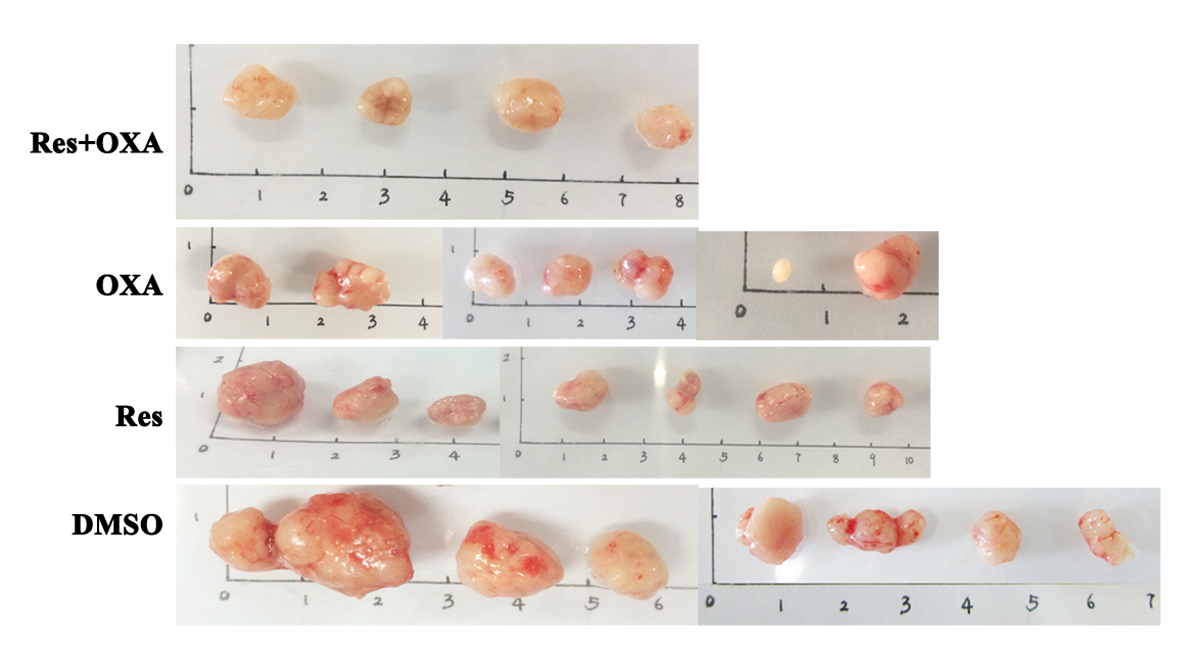

Supplement: Additional file 3: Figure S3. — HCT-116 xenografts in nude mice were collected after 2-week treatment of Res, Oxa or Res + Oxa. (TIF 3.44 mb) [file 12885_2015_1958_MOESM3_ESM.tif]

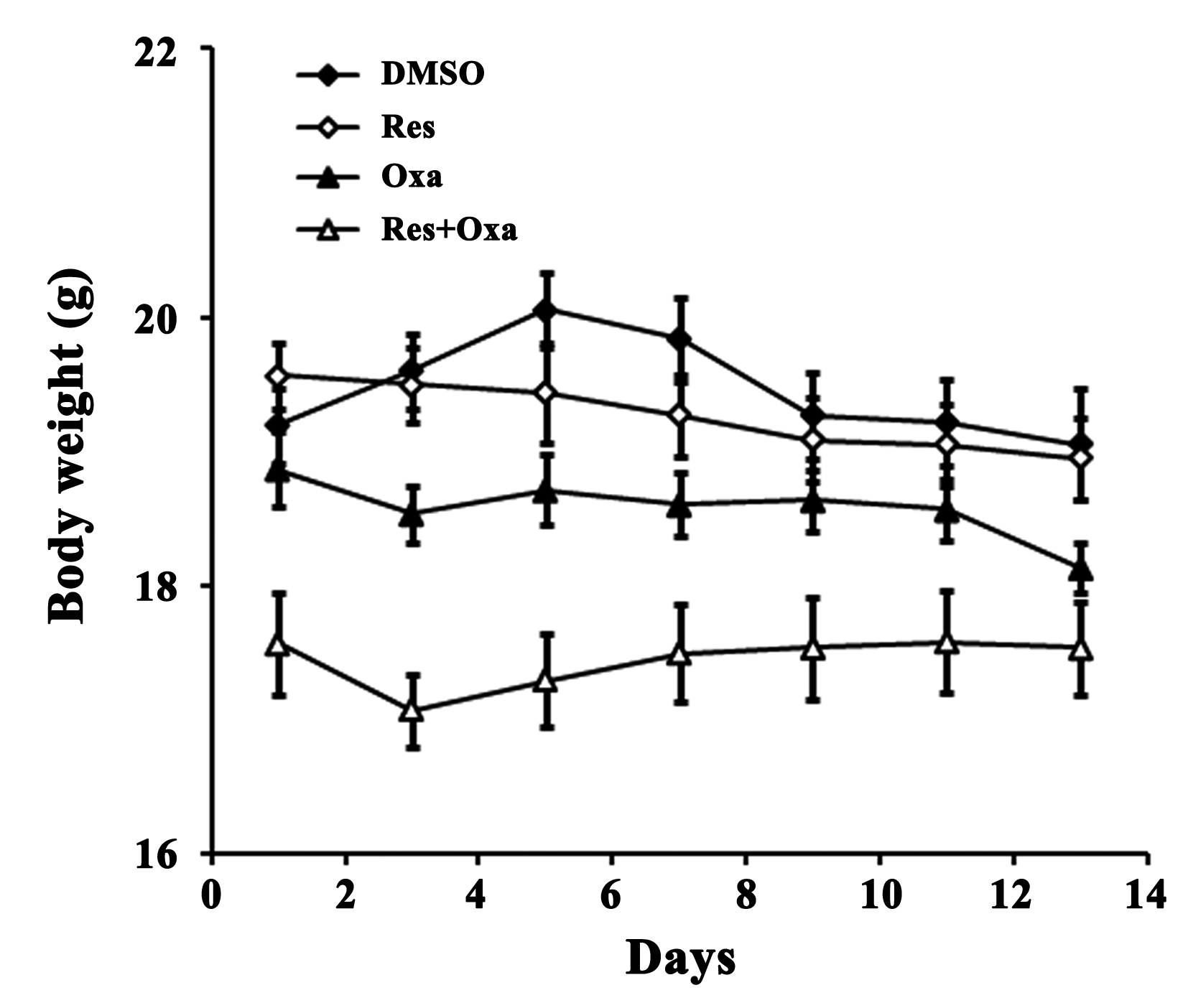

Supplement: Additional file 4: Figure S4. — There was no apparent body weight lost in any group during the animal experiment, suggesting no toxicity for mice. (TIF 2934 kb) [file 12885_2015_1958_MOESM4_ESM.tif]
